# Comprehensive Genomic Analysis for Identifying FZD6 as a Novel Diagnostic Biomarker for Acute Myeloid Leukemia

**DOI:** 10.1155/2022/9130958

**Published:** 2022-11-18

**Authors:** Li Yang, Deyu Ma, Shi Tang, Tingting Jiang, Jie Yu, Li Wang, Lin Zou

**Affiliations:** ^1^Center for Clinical Molecular Laboratory Medicine of Children's Hospital of Chongqing Medical University, Chongqing, China 400014; ^2^Department of Hematology, The First Affiliated Hospital of Chongqing Medical University, Chongqing, China 400016; ^3^National Clinical Research Center for Child Health and Disorders (Chongqing), Chongqing, China 400014; ^4^Ministry of Education Key Laboratory of Child Development and Disorders, Chongqing, China 400014; ^5^China International Science and Technology Cooperation Base of Child Development and Critical Disorders, Chongqing, China 400014; ^6^Center for Pediatric Hematology Diseases of Children's Hospital of Chongqing Medical University, Chongqing, China 400014; ^7^Clinical Research Unit, Children's Hospital of Shanghai Jiao Tong University, Shanghai, China 200062; ^8^Institute of Pediatric Infection, Immunity and Critical Care Medicine, Shanghai Jiao Tong University School of Medicine, Shanghai, China 200062

## Abstract

As a family of G protein-coupled receptors (GPCRs) with a seven-span transmembrane structure, frizzled class receptors (FZDs) play crucial roles in regulating multiple biological functions. However, their transcriptional expression profile and prognostic significance in acute myeloid leukemia (AML) are unclear. In AML, the role of FZDs was explored by performing the comprehensive analysis on the relationship between clinical characteristics and mRNA expression profiles from public databases including cBioPortal for Cancer Genomics, Gene Expression Profile Interactive Analysis (GEPIA), and Cancer Cell Line Encyclopedia (CCLE). We identified that in the majority of 27 AML cell lines, frizzled class receptor 6 (FZD6) was high-expressed. A significantly higher expression of FZD6 in AML patients was observed when compared to normal controls (*P* < 0.01). Compared with intermediate and poor/adverse risk group patients, FZD6 expressed much lower in cytogenetic favorable risk group patients (*P* < 0.0001). Patients with higher-expressed FZD6 were associated with shorter overall survival (OS) (*P* = 0.0089) rather than progression-free survival (PFS). However, the predictive effect of FZD6 on OS could be reversed by hematopoietic stem cell transplantation (HSCT). The data of gene set enrichment analysis (GSEA) demonstrated that 4 gene sets, including MYC targets, HEME metabolism, E2F targets, and UV response, were differentially enriched in the high-expression FZD6 group. To conclude, the study suggested that high expression of FZD6 might be a novel poor prognostic biomarker for AML treatment.

## 1. Introduction

As a malignant hematological disease, acute myeloid leukemia (AML) is resulted from the clonal expansion of abnormally differentiated blasts, manifesting as severe infections, anemia, hemorrhage, and organ infiltration [1]. The overall prognosis of AML is poor, about 40% of adult patients aged 60 years old or younger and 10% of patients aged over 60 years could achieve long-term survival [2]. The majority of AML patients could achieve complete remission through 1 to 2 courses of induction chemotherapy, but AML shows heterogeneity. It is essential to accurately assess the prognosis and assign postremission therapies (hematopoietic stem cell transplantation (HSCT) or consolidation chemotherapy) for patients at first remission [3]. Risk stratification is often determined by consensus guidelines. However, even low-risk patients may develop a poor prognosis, suggesting that urgent requirements for novel markers can more accurately indicate the prognosis.

Class frizzled (FZD) is one of the GPCR subfamilies, including 10 FZD isoforms denoted FZD1–10 and smoothened (SMO) in mammals. Each FZD receptor is encoded by a separate gene and contains a cysteine-rich domain (CRD), which is crucial for binding to secreted Wingless/Integrated (WNT) proteins [4]. As a main receptor for receiving WNT signals, FZDs can activate the canonical or non-canonical WNT signaling pathway through the interaction with Dishevelled (DVL) protein [5, 6], fulfilling a critical function in stem cell maintenance, cell proliferation, organ formation, cell migration, damage repair, and occurrence of human diseases [7–9]. Activated canonical WNT pathway results in *β*-catenin accumulation and nuclear translocation, subsequently promoting the transcription of WNT-related genes [10]. Noncanonical pathway refers to the pathways such as the planar cell polarity (PCP) pathway and WNT/Ca2^+^pathway that rely on WNT signal transduction but do not cause changes in soluble *β*-catenin [11]. Dysregulated expression or mutation of FZD genes and the prominent role of these molecules in cancer have been observed in various human malignant diseases [12–15].

Some studies explored the role of FZDs in hematological malignant diseases. FZD7 and FZD8 are expressed in most acute lymphoblastic leukemia (ALL) cells, while FZD3, FZD4, and FZD9 are occasionally detected. Wnt3a activates related receptors to promote the proliferation of ALL cells [16]. Meanwhile, targeting FZD7 and FZD8 can increase the drug sensitivity of multidrug-resistant ALL [17]. The relative mRNA expression levels of FZD4, FZD5, and FZD7 are upregulated in drug-mediated apoptotic chronic myeloid leukemia (CML) cells, suggesting a correlation with programmed cell death [18]. FZD4 knockdown inhibits CML progenitor growth and might increase the sensitivity of CML to Tyrosine kinase inhibitors (TKI) [19]. The expression of FZD1, especially FZD6, is progressively upregulated in the transformed chronic lymphocytic leukemia (CLL) cells, and this reveals a key role of leukemogenesis [20]. CLL shows a higher FZD3 expression than normal B cells, which indicates a less favorable clinical prognosis [21, 22]. The mRNA expression of FZD1 and its ligand Wnt3 is upregulated in mantle cell lymphoma-initiating cells, and this is possibly related to chemical resistance [23]. High-expressed FZD4 in AML blasts enhances *β*-catenin stability in myeloid progenitor cells induced by Wnt3a to regulate cell apoptosis [24]. miR-212-5p with FZD5 as a functional target has been found to be low-expressed in AML cases and cells, and it served as a tumor-suppressor gene. miR-212-5p/FZD5 is likely to become a new therapeutic target for AML [25]. In patients experiencing a relapse, FZD1 expression in blasts is significantly higher. Overexpression of FZD1 may cause drug resistance in leukemia cells, whereas silencing FZD1 may reverse multidrug resistance [26].

**Figure 1:**
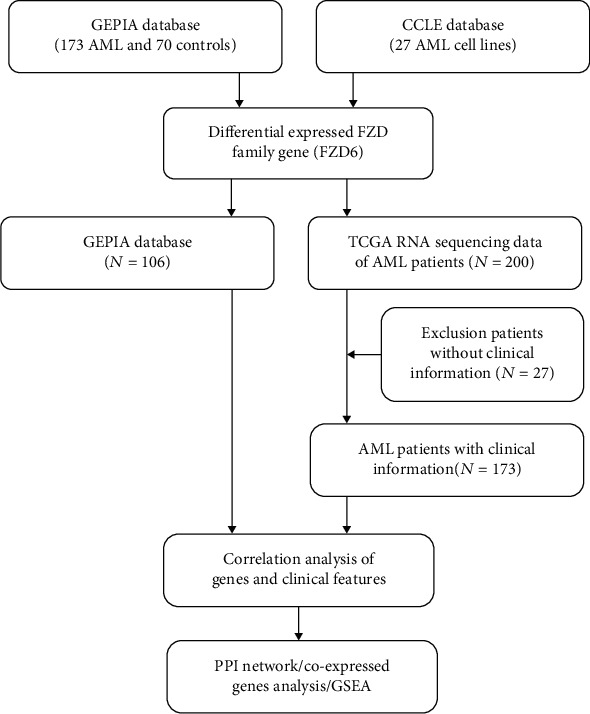
Flow chart of the study design.

Abnormal expression of the FZDs is common in AML. However, the correlation between clinical characteristics of AML and the FZDs mRNA expression profile has not been well studied. We therefore studied the transcriptional expression profiles of FZDs in AML patients using Gene Expression Profiling Interactive Analysis (GEPIA) online databases and in AML cell lines using Cancer Cell Line Encyclopedia (CCLE). Between normal control individuals and AML patients, the cBioPortal TCGA database was applied to comprehensively analyze the differences in clinical prognostic significance. The potential underlying mechanisms and biological functions of FZD6 in AML were explored by protein-protein interaction (PPI) network and gene set enrichment analysis (GSEA). Flow work was shown in Figure 1.

## 2. Materials and Methods

### 2.1. CCLE Database

The mRNA expression profiles of FZDs in AML cell lines were downloaded directly from the CCLE website (https://portals.broadinstitute.org/ccle/home) [27], which stores tumor genomics information of 1019 cell lines from individuals of various ethnicities. The mRNA expression of the ten FZDs in 27 AML cell lines was analyzed in detail.

### 2.2. GEPIA Database

GEPIA (http://gepia.cancer-pku.cn/) supports the analysis on standardized expression data of 8,587 normal samples and 9,736 tumors from GTEx and TCGA databases [28]. The GEPIA dataset was applied to analyze the differences in FZDs' transcriptional expression between normal control individuals and AML patients, and the connection between the expression level of FZDs and OS.

### 2.3. cBioPortal for Cancer Genomics Database

cBioPortal for Cancer Genomics (https://www.cbioportal.org/) supports the exploration, visualization, and analysis of multilayer clinical and cancer genome data [29]. We downloaded the mRNA expression profiles, clinical characteristics, laboratory features, and survival data of 173/200 new AML patients from the TCGA dataset on the cBioPortal website.

### 2.4. STRING and GeneMANIA Database

STRING (https://string-db.org/) could calculate and predict PPI information to generate an objective and comprehensive interaction network, including indirect (functional) and direct (physical) interactions [30]. GeneMANIA (http://www.genemania.org) predicts gene function, analyzes gene lists, and prioritizes genes for functional assays [31]. STRING and GeneMANIA datasets were used to explore the PPI network of FZD6.

### 2.5. GSEA (Gene Set Enrichment Analysis)

The underlying biological mechanism of FZD6 in AML was explored. GSEA identified the potential statistically significant differences between low FZD6 expression and high FZD6 expression groups. GSEA was performed on predefined gene sets, hallmark gene set (h.all.v7.0.symbols.gmt) from MsigDB. Statistically significant was considered when the false discovery rate (FDR) <0.05 and adjusted *P* value <0.05.

### 2.6. Statistical Analysis

Based on the median FZD6 expression level, the patients were grouped into two groups according to low- and high-expression of FZD6. All statistical analyses were carried out in SPSS23.0 software (SPSS Inc, Chicago, IL, USA) and GraphPad Prism5 (GraphPad Software Inc, La Jolla, CA, USA). The relationship between FZD6 expression and clinical features was analyzed by the chi-squared test or Fisher's exact test. Survival curves were plotted by the Kaplan-Meier method, and the survival difference between the two groups of patients was analyzed by log-rank test. Multivariate analyses were conducted with Cox proportional hazard model. *P* value <0.05 was defined as of statistical significance.

## 3. Results

### 3.1. mRNA Expression Profiles of FZDs in AML Cell Lines and AML Patients

**Figure 2:**
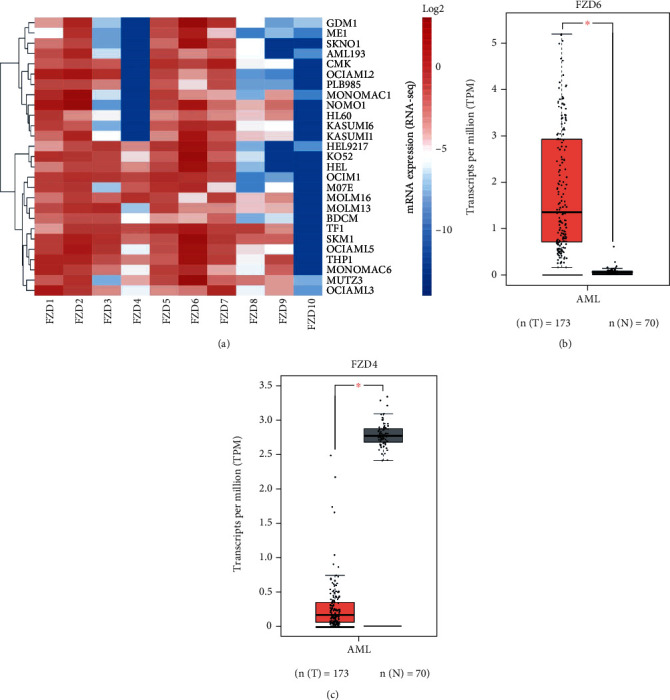
mRNA expression profiles of FZDs in AML cell lines and AML patients. (a) Heat map of FZD1 to FZD10 expression in 27 AML cell lines. (b) Expression of FZD6 in AML patients (*n* = 173) compared to normal samples (*n* = 70) in TCGA and GTEx dataset. (c) Expression of FZD4 in AML patients (*n* = 173) compared to normal samples (*n* = 70) in TCGA and GTEx dataset (^∗^*P* < 0.01).

The mRNA expression of FZDs was determined in 27 AML cell lines from the CCLE database. High mRNA expression of FZD1, FZD2, FZD5, FZD6, and FZD7 were detected in AML cell lines. FZD6 had the highest expression, and FZD10 had the lowest expression in the cells. FZD4 showed a polarized expression (Figure 2(a)). To further investigate the mRNA expression profiles of FZDs in AML patients, we explored their mRNA expression using the GEPIA online database. It has been found that FZD6 was remarkably higher-expressed (*P* < 0.01) in AML patients compared with that in normal controls (Figure 2(b)). FZD4 was significantly lower-expressed (*P* < 0.01) in AML patients compared with that in normal individuals (Figure 2(c)). Other FZDs did not exhibit obvious differences in their mRNA expression levels (*P* > 0.05) (Figure S1). High-expressed FZD6 in AML patients was consistent with that in the cell lines. However, low-expressed FZD4 was different from that in cell lines. Therefore, only FZD6 was included in the follow-up correlation analysis with clinical features.

### 3.2. High FZD6 Expression Is Related to Poorer Risk Classification in AML Patients

**Figure 3:**
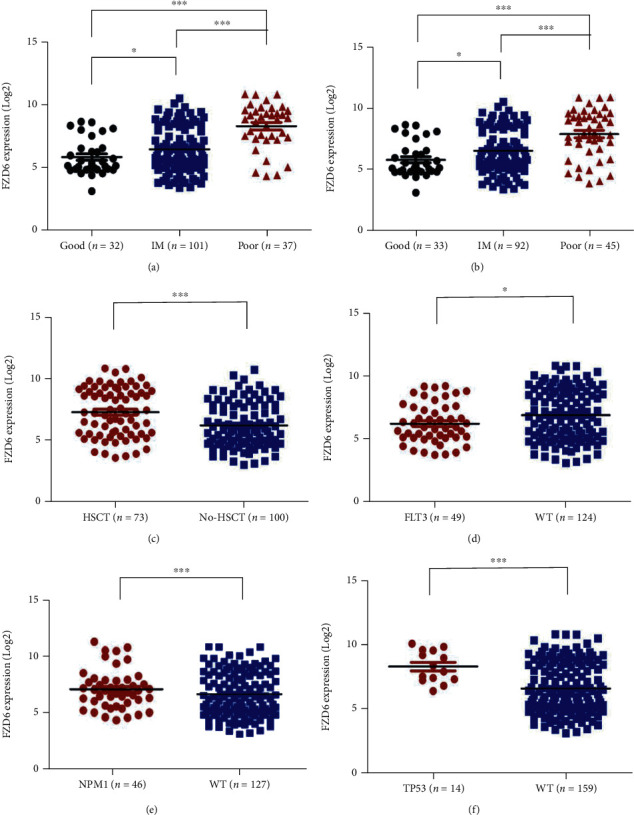
High FZD6 expression is related to poorer risk classification in AML patients. (a) FZD6 expression differences among low risk (*n* = 32), intermediate (IM) risk (*n* = 101), and poor risk (*n* = 37), according to CRC. (b) FZD6 expression differences among good risk (*n* = 33), intermediate (IM) risk (*n* = 92), and adverse risk (*n* = 45), according to ELN risk stratification. (c) FZD6 expression differences between patients received HSCT (*n* = 73) or did not received HSCT (no-HSCT) (*n* = 100). (d) FZD6 expression differences between patients had FLT3 mutation (*n* = 49) or not (*n* = 124). (e) FZD6 expression differences between patients had NPM1 mutation (*n* = 46) or not (*n* = 127). (f) FZD6 expression differences between patients had TP53 mutation (*n* = 14) or not (*n* = 159). ^∗^*P* < 0.05; ^∗∗^*P* < 0.01; ^∗∗∗^*P* < 0.001.

The correlation of clinical characteristics with FZD6 mRNA expression in AML patients was analyzed, as shown in the workflow (Figure S2). From the cBioPortal TCGA database, clinical characteristics of AML patients and the mRNA expression data of FZD6 were downloaded. Based on the median FZD6 expression level, all patients (*n* = 173) with clinical information and RNA sequencing data were grouped into the low FZD6 expression group (*n* = 87) and high FZD6 expression group (*n* = 86) [32]. No significant differences were detected between the two groups in terms of sex, age, peripheral blood (PB) blasts, white blood cell (WBC) count, and induction chemotherapy regimens (intensive regimens or nonintensive regimens) (*P* > 0.05) (Table 1, Figures S3(a)–S3(c). Our data showed that low FZD6 expression patients had a higher percentage of bone marrow (BM) blasts (*P* = 0.0014) (Table 1). According to the cytogenetic risk stratification (CRC) and European Leukemia Net (ELN) risk stratification, the proportion of patients with intermediate-risk and poor-/adverse-risk was higher in the high FZD6 expression group than in the low FZD6 expression group (*P* < 0.001) (Table 1). Meanwhile, FZD6 expression was significantly upregulated and was accompanied by elevated CRC and ELN risk stratification (Figures 3(a), 3(b)). Additionally, more patients in the high FZD6 expression group were treated by HSCT (*P* = 0.003) (Table 1). Correspondingly, patients who received HSCT showed much higher FZD6 expression compared with those who received chemotherapy or no treatment (*P* < 0.0001) (Figure 3(c)). Further exploration showed that in patients receiving different types of allogeneic hematopoietic stem cell transplantation (allo-HSCT), there were no differences in FZD6 expression level, but the level was higher than in patients with chemotherapy or without treatment (Figure S3(d)). According to FAB (French-American-British) classification, the expressions of FZD6 in AML-M3 patients (a highly curable subtype of AML) were significantly lower than in other types (Figure S3(e)).

Furthermore, we explored the relationship between common gene mutations in AML and FZD6 expression. The incidence analysis of FLT3, TP53, DNMT3A, NPM1, RUNX1, ASXL1, IDH1, and IDH2 mutations in the FZD6 high-expression group and the FZD6 low-expression group showed that the FZD6 low-expression group had a greater possibility of NPM1 mutation (*P* < 0.001) (Table 1). TP53 mutations, which independently affect prognosis, were higher in the high-expression group (*P* < 0.001) (Table 1). The expression level of FZD6 in the FLT3 mutation group (*P* = 0.0186) and NPM1 mutation group (*P* < 0.0001) was lower (Figure 3(d)–3(e)), while that of FZD6 in the TP53 mutation group was higher (*P* = 0.0001) (Figure 3(f)). The relationship between FZD6 expression and other gene mutations was not statistically significant (Table 1) (Figures S3(f)–S3(j)). Given that the FLT3 mutation rate and the coexistence of NPM1 mutations could affect prognosis, the expression of FZD6 is closely related to TP53 mutation, which suggests a poor prognosis.

### 3.3. High FZD6 Expression Is Related to Poor OS in AML Patients

**Figure 4:**
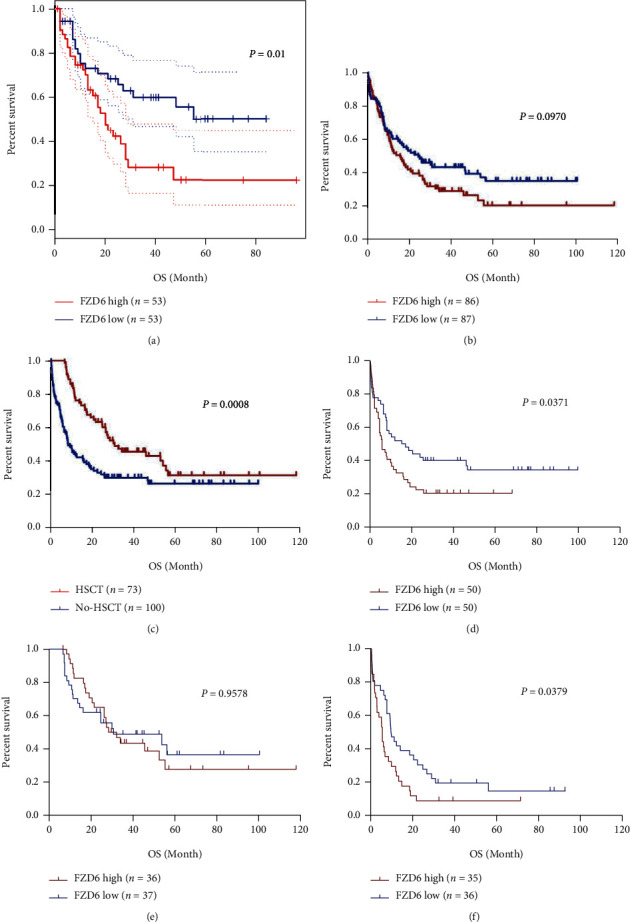
High FZD6 expression is related to poor OS in AML patients. (a) The association between FZD6 and OS of AML patients on the GEPIA website. (b) The association between FZD6 and OS of AML patients based on the cBioPortal TCGA database. (c) The association between HSCT and OS of AML patients. (d) The association between FZD6 and OS of AML patients did not receive HSCT. (e) The association between FZD6 and OS of AML patients received HSCT. (f) The association between FZD6 and OS of AML patients with intermediate/adverse risk did not receive HSCT.

The association of the prognosis of AML patients with FZD6 was explored. The GEPIA was applied to analyze the Kaplan-Meier survival curve of AML patients' OS. A shorter OS of AML patients with higher FZD6 expression than those with lower-expressed FZD6 was observed (*P* = 0.0089) (Figure 4(a)). Then, we analyzed the association of FZD6 expression with OS and progression-free survival (PFS) based on data downloaded from the cBioPortal TCGA database. Patients in the high FZD6 expression group showed a shorter OS but without statistical significance (median: 15.80 vs. 24.80 months; *P* = 0.097) (Figure 4(b)). As HSCT is an important treatment option, we divided 173 AML patients into HSCT group (*n* = 73) and no-HSCT group (*n* = 100), and then analyzed the relationship between FZD6 level and prognosis of patients in each group. The results showed that the OS in patients without HSCT treatment was significantly reduced (median: 30.60 vs. 8.20 months; *P* = 0.0008) (Figure 4(c)). In the group of patients without HSCT treatment, higher FZD6 expression patients had a shorter OS compared with those with lower FZD6 expression (median: 5.70 vs. 16.20 months; *P* = 0.037) (Figure 4(d)). However, no statistical differences were detected in the patient group who received HSCT (median: 32.30 vs. 30.60 months; *P* = 0.9578) (Figure 4(e)). Except for low-risk patients who generally did not need HSCT, among the intermediate-/adverse-risk patients with HSCT, a high FZD6 expression group showed a significantly shorter OS than that of low FZD6 expression group (median: 4.55 vs. 8.05 months; *P* = 0.0379), indicating that high FZD6 expression was correlated with unfavorable prognosis (Figure 4(f)). No similar results on the relationship between FZD6 expression and PFS were observed (Figure S4(a)–(d)).

In addition to FZD6 expression, as shown by Cox regression analyses, other clinical factors may also affect the prognosis of AML. Age (hazard ratio (HR) = 1.018, 95% CI: 1.001-1.035, *P* = 0.040), WBC counts (HR = 1.007, 95% CI: 1.002-1.011, *P* = 0.040), ELN risk stratification (HR = 2.720, 95% CI: 1.341-5.518, P = 0.006), induction therapy (HR = 0.371, 95% CI: 0.211-0.654, *P* = 0.001), HSCT (HR = 0.508, 95% CI: 0.304-0.848, *P* = 0.010), and FZD6 expression (HR = 1.264, 95% CI: 1.025-1.559, *P* = 0.028) were the independent prognosis factors of OS in AML patients. However, only WBC counts (HR = 1.007, 95% CI: 1.002-1.011, *P* = 0.006) and ELN risk stratification (HR = 2.521, 95% CI: 1.173-5.419, *P* = 0.018) could predict the PFS of AML patients (Table 2).

### 3.4. Potential Role of FZD6 in AML

**Figure 5:**
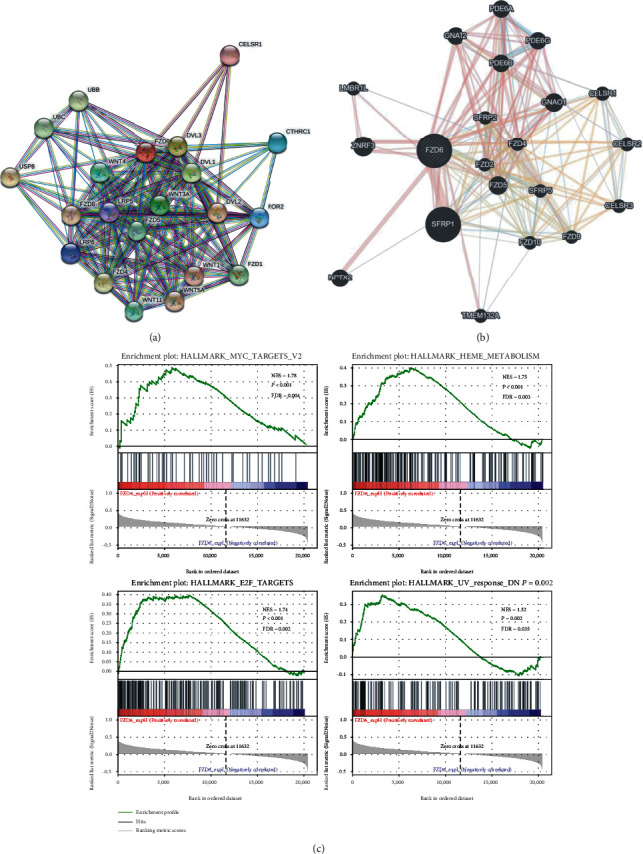
Potential role of FZD6 in AML. (a) PPI network of FZD6 analyzed by STRING. (b) PPI network of FZD6 analyzed by GeneMANIA. (c) GSEA analysis of AML patients based on FZD6 expression.

To explore the core regulatory genes and underlying mechanisms, we used the STRING and GeneMANIA databases to construct a PPI network. The results demonstrated that FZD6 interacted with the protein related to canonical and noncanonical WNT signaling pathways, which was in line with its physiological function (Figures 5(a) and 5(b)). Based on the cBioPortal dataset, 266 coexpressed genes are shown in Table S1 (Spearman's correlation>0.4 and *P* value <0.05), and the top 10 positively coexpressed genes and negatively coexpressed genes are shown in Table 3.

Furthermore, to explore underlying mechanisms, we compared the transcriptomes of the high FZD6 expression group and the low FZD6 expression groups based on the cBioPortal TCGA database. A total of 1152 genes were identified, including 386 upregulated and 766 downregulated. Statistical differences between high FZD6 and low FZD6 groups were found (*P* ≤ 0.05, ∣log2FC | ≥1) (Figure S5(a)). The Gene Ontology (GO) analysis and the top 20 enriched Kyoto Encyclopedia of Genes and Genomes (KEGG) pathways are shown in Figures S5(b) and S5(c). GSEA was used to analyze the differences between high and low FZD6 expression data sets for screening gene sets involved in AML. Our result demonstrated that 4 gene sets, including HEME metabolism (NES = 1.75, FDR = 0.003, *P* < 0.001), UV response (NES = 1.52, FDR = 0.035, *P* = 0.002), E2F targets (NES = 1.74, FDR = 0.002, *P* < 0.001), MYC targets (NES = 1.78, FDR = 0.004, *P* < 0.001), were differentially enriched in the high FZD6 expression group. All the 4 sets were critical for AML development and progression [33–36]. The results pointed to a potential function of FZD6 in AML development (Figure 5(c)).

## 4. Discussion

The clinical outcome of AML is highly heterogeneous. In particular, the prognosis and postremission treatment of AML is based on risk stratification. It is a great challenge for clinicians that patients with the same risk stratification may have completely different prognoses. Thus, finding novel biomarkers for prognostic evaluation is an urgent task. Combined analysis using the databases of CCLE, GEPIA, cBioPortal, STRING, and GeneMANIA databases manifested high-expressed FZD6 in AML cell lines and patients, which was positively correlated with the risk factors, OS and HSCT, and that 4 gene sets (MYC targets, HEME metabolism, E2F targets, and UV response) were differentially enriched in the high FZD6 expression group.

FZDs serve as receptors for secreted WNT ligands in the WNT signaling pathway and play crucial roles in regulating multiple biological functions [37]. Their transcriptional expression profile and prognostic significance in AML remain relatively unknown. Although we measured all members of the FZD family in the AML cell lines, only the expression of FZD1, FZD2, FZD5, FZD6, and FZD7 were higher, with FZD6 being the highest. Therefore, we further explored the correlation of the clinical characteristics of AML with FZD6 expression.

High FZD6 expression has been identified in various cancer cells, including in glioblastoma, oral squamous cell carcinoma, and pancreatic adenocarcinoma, showing a strong correlation with tumor malignancy and prognosis [38–40]. At the same time, we also used SangerBox (http://vip.sangerbox.com) to evaluate the relationship between the expression and prognosis of FZD6 in pan-cancer, and the results were consistent with the previous literature (Figure S6) [39, 40]. In hematopoietic malignancies, WNT signaling activated by WNT10B/FZD6 promotes intracellular effectors and leukemic expansion in WNT10BR-positive T-ALL cells [41]. WNT/FZD (especially FZD6) self-renewal signals are pathologically reactivated in the neoplastic transformation of mature B cells in CLL [20]. lncRNA prostate cancer-associated transcript-1(PCAT-1) interacts with FZD6 to activate WNT/*β*-catenin signaling and may exert a crucial effect on AML pathogenesis [42]. Currently, no relevant studies were conducted to explore the association between FZD6 and the clinical features of AML.

We detected that high FZD6 expression was associated with poor cytogenetic risk classification, adverse ELN risk stratification, TP53 mutation, and short survival of AML patients, according to several powerful publicly available datasets. No statistically significant difference has been found between FZD6 expression level and OS of AML patients. However, when excluding AML patients who received HSCT, patients with a higher FZD6 expression had a shorter OS. This was possibly due to the fact that HSCT as a potentially curative treatment option for AML was the most effective prognosis protection factor, which may partially interfere with the effect of FZD6 expression on prognosis. The results indicated that the predictive effect of FZD6 on OS could be reversed by HSCT, and that intermediate/adverse risk patients with higher FZD6 showed a more urgent need for HSCT. Multivariate survival analysis demonstrated that older age, higher WBC counts, intermediate/adverse risk, and higher FZD6 expression are independent prognostic factors for poor OS, while intensive chemotherapy and HSCT are protective factors for the prognosis [43, 44]. Taken together, FZD6 may become a new prognostic biomarker for AML.

Moreover, mediator of the WNT canonical ligands binding to FZD6 promotes *β*-catenin accumulation and nuclear translocation and activates downstream target genes, such as Cyclin D1, c-MYC, Survivin, and MMP, thereby regulating cell proliferation [45]. However, most reports indicate a prevalent role in the noncanonical pathway [46]. PCP signaling pathway sequentially activates Rac and Rho small GTPases and JNK and promotes actin polymerization and cytoskeleton modification. WNT/Ca^2+^ pathway activates calcineurin and the nuclear factor of activated T cells (NFAT) transcription factors, which regulate transcriptional programs involved in cell fate and cell migration [47]. To further understand the functional roles of FZD6, we analyzed the FZD6 PPI network and generated a gene network using STRING and GeneMANIA databases. The results showed that FZD6 mainly interacted with proteins involved in the WNT signaling pathway, such as WNT proteins, DVL proteins, secreted frizzled-related protein 1 (SFRP1), neuronal pentraxin 2 (NPTX2), and zinc and ring finger 3 (ZNRF3), and so on. Moreover, the effect of FZD6 on AML resulted from the activation of the WNT signaling pathway, therefore showing a prognostic significance in AML [48]. Then, we explored the FZD6 coexpression network, and the data suggested that FZD6 may participate in other signaling pathways and activate its underlying molecular mechanisms to exert its effect on AML.

There were several differentially expressed genes in the high FZD6 expression group and the FZD6 low-expression group. GO analysis and KEGG enrichment analysis of these genes do not show specific function and pathway changes. We speculated that FZD6 exerted its biological function through the gene set analyzed by GSEA. High-expressed FZD6 was mainly involved in 4 gene sets, including MYC targets, HEME metabolism, E2F targets, and UV response, as shown by GSEA analysis. This provided a potential direction to explore its biological functions in AML patients. MYC target gene network is estimated to account for about 15% of all human genes, involving in metabolism, mitochondrial function cell cycle regulation, protein synthesis, and ribosome biogenesis [49]. Previous research had shown that higher MYC expressions are related to poorer survival outcomes and contribute to the chemo-resistance of AML [33]. Consistently, in our study, high-expression of FZD6 can activate the WNT signaling pathway and promote the expression of MYC gene, resulting in a poor prognosis. Crosstalk between metabolic and survival pathways is critical for cellular homeostasis. Heme biosynthesis, which could reduce apoptosis through electron transport chain (ETC) activation, has been seen as an apoptotic modulator in AML [34]. As a survival-enhancing molecule of AML, Heme oxygenase-1 (HO-1) promotes tumor progression, carcinogenesis, and chemical resistance [50]. At the same time, E2F expression and/or elevated E2F target expression in tumors could cause uncontrolled proliferation and were linked to a poor prognosis [35]. UV response mainly reflects DNA damage [51]. Recent findings highlighted that increased DNA damage and abnormal DNA damage response (DDR) were the key features of AML blasts [36], which would affect susceptibility, disease progression, and resistance to standard chemotherapy [52]. These may explain the effect of FZD6 on AML, but the specific mechanisms required further exploration.

The limitations of this study need to be equally acknowledged. Firstly, our study mainly explored the transcriptional levels of FZDs in AML and their association with prognosis. However, as GPCRs, the protein levels of FZDs are closely related to their function, which requires further exploration. This study did not verify the biological function and related mechanism of FZD6 in vitro and in vivo, and in-depth research is needed. In addition, AML patients show heterogeneity, and the one-sidedness of a single index can only provide a certain clinical reference, which requires comprehensive evaluation.

## 5. Conclusions

To conclude, we comprehensively analyzed the mRNA expression profiles of FZDs in AML patients and 27 AML cell lines through different online resources. Our results indicated that FZD6 was the only overexpressed molecule in FZDs of AML patients, and that high-expressed FZD6 was associated with poor/adverse risk stratification. Furthermore, FZD6 was a potential independent adverse survival factor in patients, but the predictive effect on OS could be reversed by HSCT. Overall, here we showed that the effect of FZD6 on AML may be derived from the activation of the WNT signaling pathway, and the underlying molecular mechanism demands further illustration. Our findings helped clinicians better understand the role of FZDs in AML and highlighted FZD6 as a candidate gene for AML prognosis. FZD6 is also expected to become a new therapeutic target, but this should be confirmed in future studies before direct application.

## Figures and Tables

**Table 1:** Correlation of FZD6 expression with clinical characteristics in AML patients.

	FZD6 high (*n* = 86)	FZD6 low (*n* = 87)	*P*
Sex, *n* (%)			0.490
Female	38 (44.2)	43 (49.4)	
Male	48 (55.8)	44 (50.6)	
Age, years			0.818
Median (range)	58 (21-88)	58 (18-82)	
WBC (×10^9^/L)			0.985
Median (range)	14.7 (0.5-223.8)	14.5 (0.4-297.4)	
BM blasts (%)			0.001
Median (range)	67 (30-100)	78 (32-100)	
PB blasts (%)			0.099
Median (range)	48 (0-97)	25 (0-98)	
CRC, *n* (%)^∗^			<0.001
Favorable	9 (10.7)	23 (26.7)	
Intermediate	44 (52.4)	57 (66.3)	
Poor	31 (36.9)	6 (7.0)	
ELN risk stratification, *n* (%) ^∗^			<0.001
Favorable	9 (10.7)	24 (27.9)	
Intermediate	41 (48.8)	51 (59.3)	
Adverse	34 (39.5)	11 (13.0)	
Induction therapy, *n* (%) #			0.910
Intensive	66 (80.5)	69 (81.2)	
Nonintensive	16 (19.5)	16 (18.8)	
HSCT, *n* (%)			0.003
Yes	46 (53.5)	27 (31.0)	
No	40 (46.5)	60 (69.0)	
Gene mutation			
FLT3, *n* (%)	20 (23.3)	29 (33.3)	0.141
TP53, *n* (%)	13 (15.1)	1 (1.2)	<0.001
DNMT3A, *n* (%)	23 (26.7)	19 (21.8)	0.451
NPM1, *n* (%)	9 (10.5)	37 (42.5)	<0.001
RUNX1, *n* (%)	10 (11.6)	5 (5.8)	0.188
ASXL1, *n* (%)	3 (3.49)	0 (0)	0.121
IDH1, *n* (%)	9 (10.5)	8 (9.2)	0.804
IDH2, *n* (%)	9 (10.5)	7 (8.1)	0.611

^∗^The total patient number is 170 for three patients are lacking evaluable cytogenetic or molecular Information. #Total patient number is 167 for six patients who did not receive any treatment after diagnosis. Intensive treatment means the induction therapy regimen is abased on 7 + 3 regimens. Nonintensive treatment means epigenetic therapy and low-intensive treatment. Abbreviations: WBC, white blood cell; BM, bone marrow; PB, peripheral blood; CRC, cytogenetic risk classification; ELN, European Leukemia Net; HSCT, hematopoietic stem cell transplantation.

**Table 2:** Cox proportional hazards model for OS and PFS in AML patients.

Variables	OS		PFS	
	HR (95% CI)	*P*	HR (95% CI)	*P*
Age	1.018 (1.001-1.035)	0.040	1.008 (0.991-1.026)	0.335
Sex (male vs. female)	0.811 (0.547-1.203)	0.298	0.752 (0.480-1.177)	0.212
WBC counts	1.007 (1.002-1.011)	0.002	1.007 (1.002-1.011)	0.006
BM blasts percentage	1.009 (0.998-1.020)	0.127	1.002 (0.989-1.016)	0.769
ELN risk stratification		0.006		0.018
(intermediate/adverse vs. favorable)	2.720 (1.341-5.518)		2.521 (1.173-5.419)	
Induction therapy		0.001		0.474
(intensive vs. nonintensive)	0.371 (0.211-0.654)		0.736 (0.318-1.702)	
HSCT (yes vs. no)	0.508 (0.304-0.848)	0.010	1.149 (0.644-2.052)	0.637
FZD6 expression	1.264 (1.025-1.559)	0.028	1.081 (0.853-1.370)	0.518

Abbreviations: OS, overall survival; PFS, progression free survival; HR, hazard ratio; CI, confidence interval.

**Table 3:** Top 10 positively and negatively coexpressed genes of FZD6.

Correlated gene	Cytoband	Co-ex relationship	Spearman's correlation	*P*	*q* value
MLLT3	9p21.3	Positively	0.576	1.17*E* − 16	1.42*E* − 12
SPAG16	2q34	Positively	0.574	1.44*E* − 16	1.42*E* − 12
AKAP6	14q12	Positively	0.558	1.50*E* − 15	9.88*E* − 12
PRKCH	14q23.1	Positively	0.549	4.92*E* − 15	2.43*E* − 11
MREG	2q35	Positively	0.535	3.51*E* − 14	1.39*E* − 10
ATP8A1	4p13	Positively	0.528	7.94*E* − 14	2.61*E* − 10
ARMCX5	Xq22.1	Positively	0.525	1.21*E* − 13	3.09*E* − 10
KLHL6	3q27.1	Positively	0.525	1.25*E* − 13	3.09*E* − 10
RAB39B	Xq28	Positively	0.522	1.74*E* − 13	3.81*E* − 10
TRAF5	1q32.3	Positively	0.521	2.10*E* − 13	4.06*E* − 10
PARL	3q27.1	Negatively	-0.492	6.47*E* − 12	3.65*E* − 09
BTG1	12q21.33	Negatively	-0.49	8.02*E* − 12	4.05*E* − 09
CFD	19p13.3	Negatively	-0.485	1.35*E* − 11	6.12*E* − 09
RPA4	Xq21.33	Negatively	-0.481	2.08*E* − 11	8.73*E* − 09
HOMER3	19p13.11	Negatively	-0.475	4.07*E* − 11	1.54*E* − 08
RAC1	7p22.1	Negatively	-0.462	1.57*E* − 10	4.24*E* − 08
PPP1R27	17q25.3	Negatively	-0.46	2.00*E* − 10	4.92*E* − 08
RNASE2	14q11.2	Negatively	-0.453	4.06*E* − 10	8.90*E* − 08
ZNHIT1	7q22.1	Negatively	-0.451	4.96*E* − 10	1.05*E* − 07
CST3	20p11.21	Negatively	-0.449	5.87*E* − 10	1.21*E* − 07

## Data Availability

The data used to support the findings of this study have been deposited in the CCLE (https://portals.broadinstitute.org/ccle/home), CEPIA (http://gepia.cancer-pku.cn/), cBioPortal for Cancer Genomics (https://www.cbioportal.org/), STRING (https://string-db.org/),and GeneMANIA (http://www.genemania.org) repositories.

## Supplementary Materials

Supplementary 1 Supplementary Figure 1: expression of FZD1 to FZD10 in AML patients (*n* = 173) compared to normal samples (*n* = 70) in TCGA and GTEx dataset.

Supplementary 2 Supplementary Figure 2: flowchart for extraction of database.

Supplementary 3 Supplementary Figure 3: (a) FZD6 expression differences between patients ≥60 years old and < 60 years old; (b) FZD6 expression differences between males and females; (c) FZD6 expression differences between WBC < 10 × 10^9^/L and WBC ≥ 10 × 10^9^/L; (d) FZD6 expression differences in patients who received different types of HSCT and no-HSCT; (e) FZD6 expression difference in FAB classifications; (f) FZD6 expression differences between patients had DMNT3A mutation (*n* = 42) or not (*n* = 131); (g) FZD6 expression differences between patients had RUNX1 mutation (*n* = 15) or not (*n* = 158). (h) FZD6 expression differences between patients had ASXL1 mutation (*n* = 3) or not (*n* = 170). (i) FZD6 expression differences between patients had IDH1 mutation (*n* = 17) or not (*n* = 156). (j) FZD6 expression differences between patients had DMNT3A mutation (*n* = 16) or not (*n* = 157). ^∗^*P* < 0.05; ^∗∗^*P* < 0.01; ^∗∗∗^*P* < 0.001.

Supplementary 4 Supplementary Figure 4: (a) the association between FZD6 and EFS of AML patients; (b) the association between HSCT and EFS of AML patients; (c) the association between FZD6 and EFS of AML patients did not receive HSCT; (d) the association between FZD6 and EFS of AML patients who received HSCT.

Supplementary 5 Supplementary Figure 5: (a) volcano plot of different gene-expression profiles between the high FZD6 and low FZD6 groups; (b) GO analysis associated with FZD6 expression; (c) top 20 KEGG pathway associated with FZD6 expression.

Supplementary 6 Supplementary Figure 6: the relationship between the expression of FZD6 and prognosis in pan-cancer.

Supplementary 7 Supplementary Table 1: coexpressed genes of FZD6 based on the cBioPortal dataset.
