# Supplementary figures and images for: Comprehensive Genomic Analysis for Identifying FZD6 as a Novel Diagnostic Biomarker for Acute Myeloid Leukemia
Source: Comput Math Methods Med. 2022 Nov 18;2022:9130958. doi: 10.1155/2022/9130958 (PMC9704059; doi:10.1155/2022/9130958)

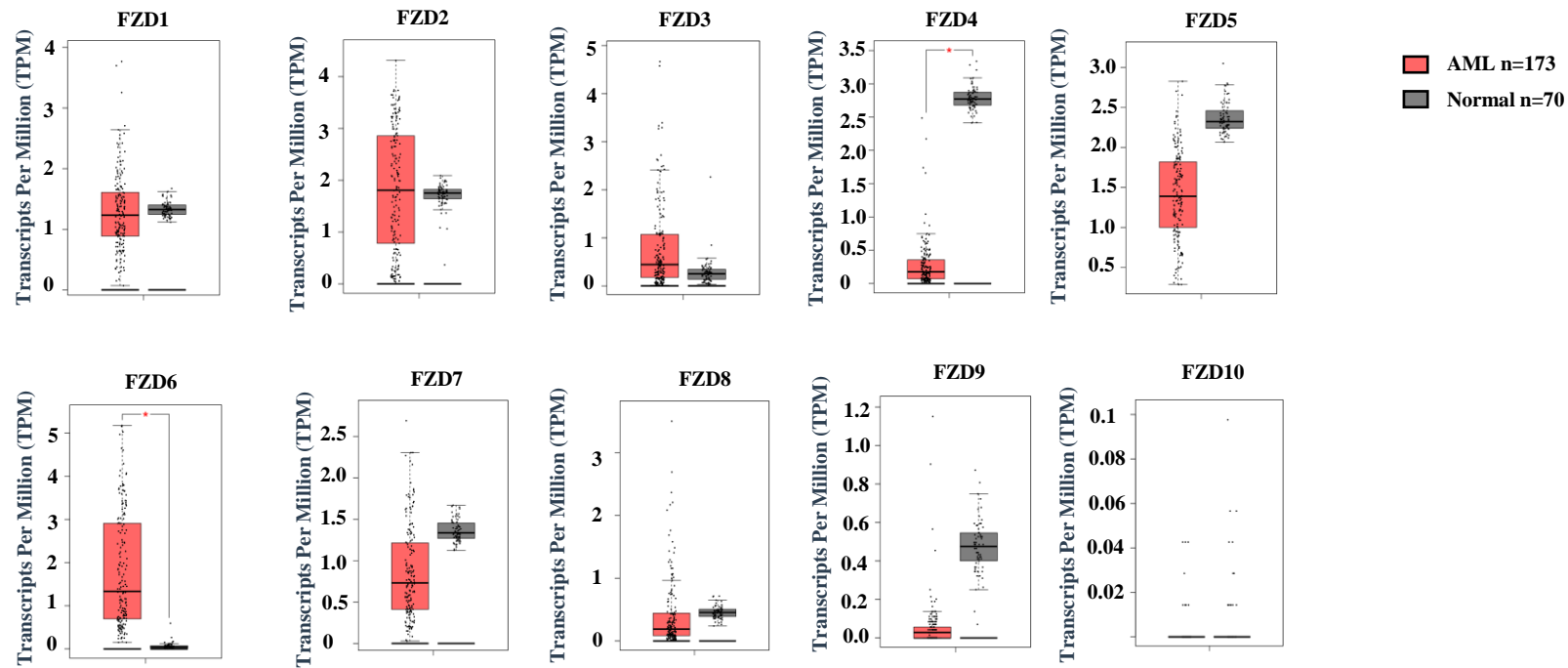

Supplement: Supplementary 1 — Supplementary Figure 1: expression of FZD1 to FZD10 in AML patients (n = 173) compared to normal samples (n = 70) in TCGA and GTEx dataset. [file 9130958.f1.pdf]

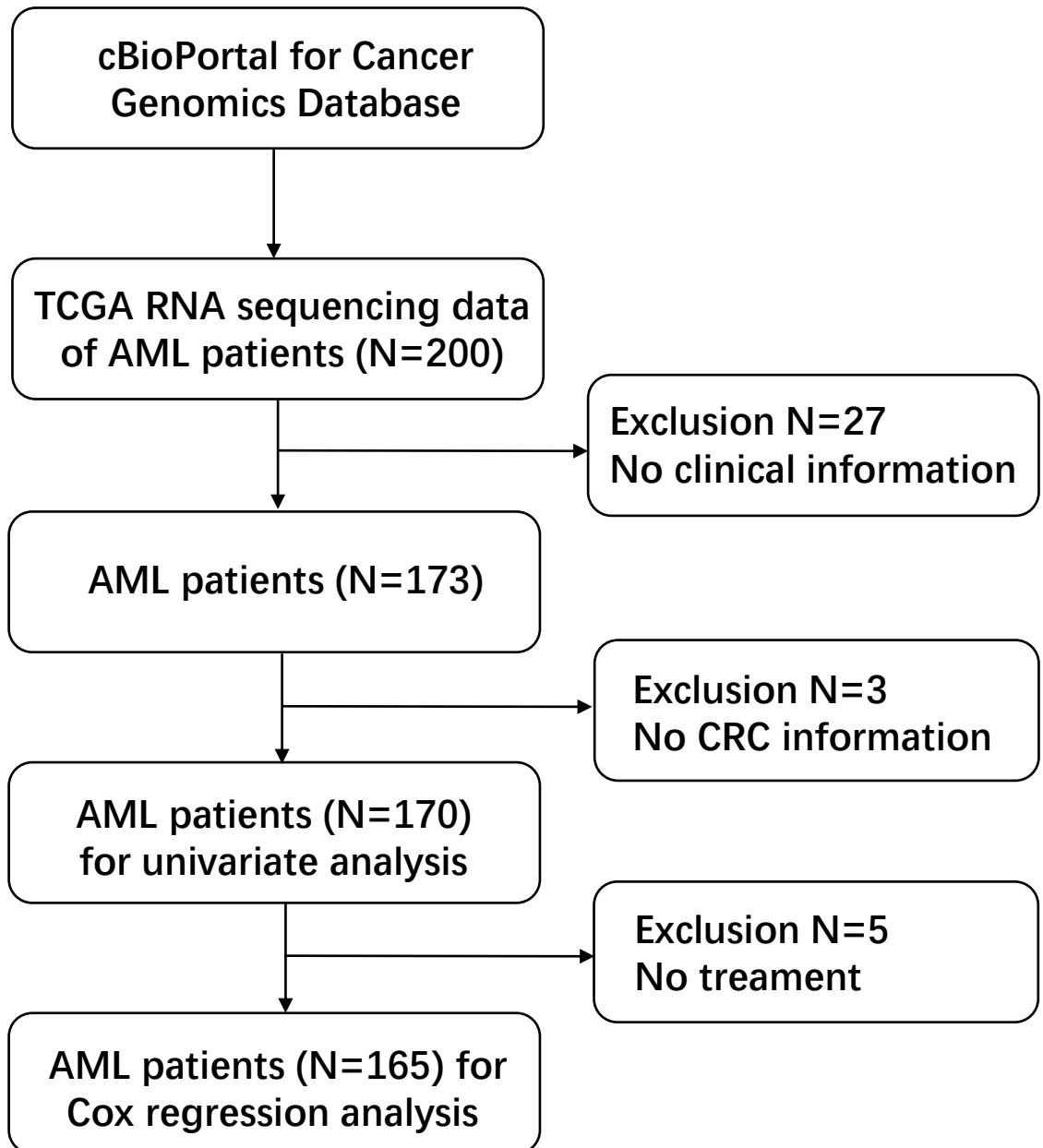

Supplement: Supplementary 2 — Supplementary Figure 2: flowchart for extraction of database. [file 9130958.f2.pdf]

**A**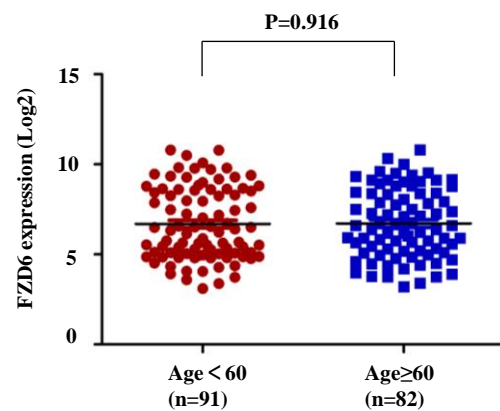**B**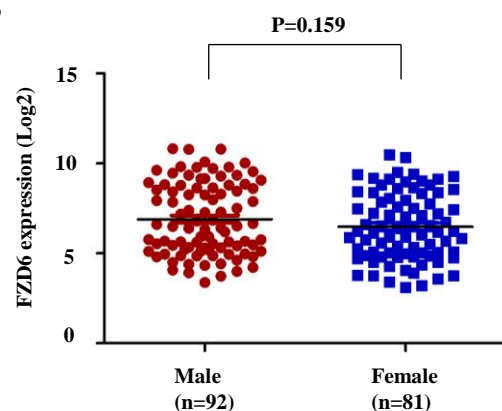**C**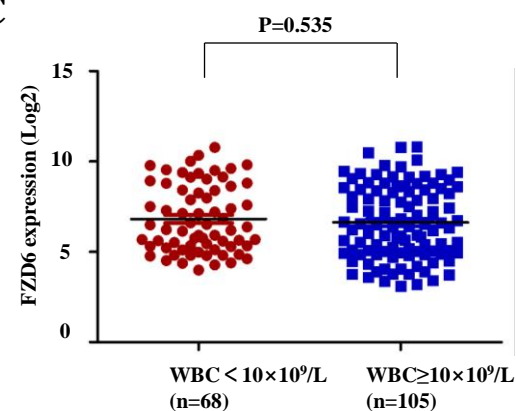**D**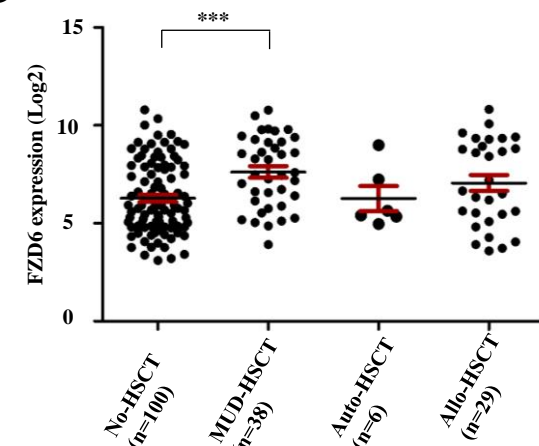**E**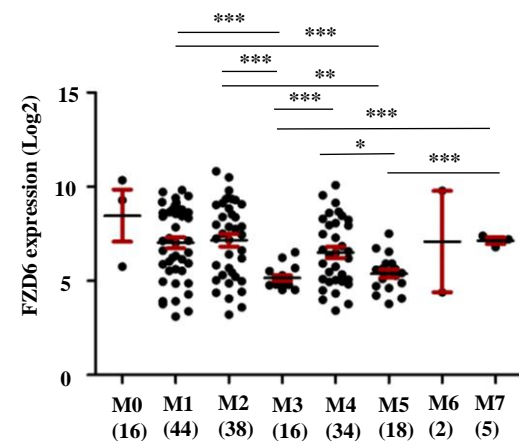**F**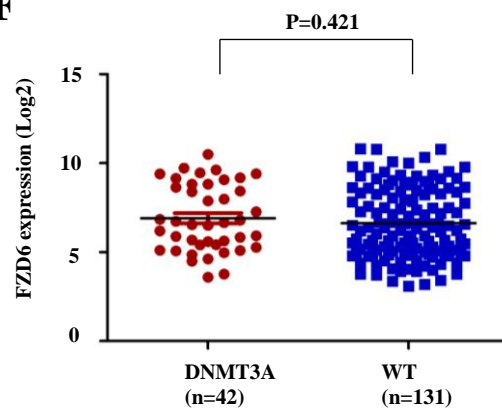**G**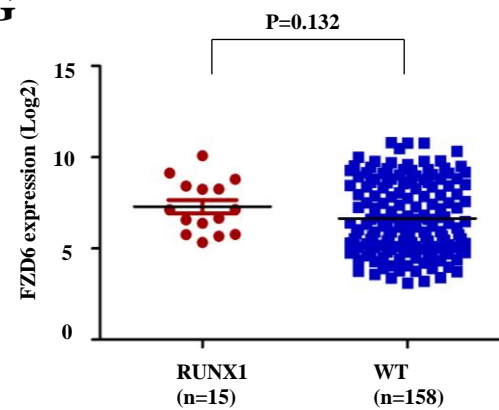**H**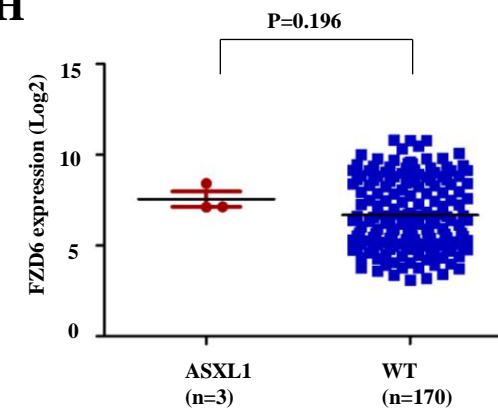**I**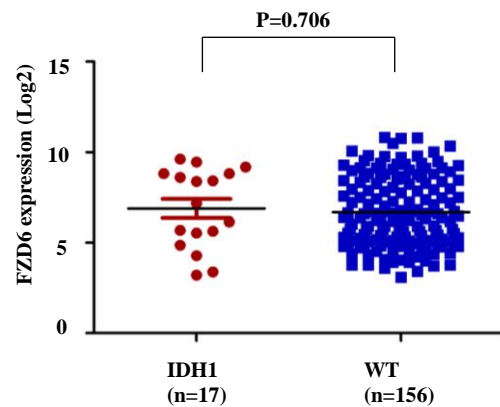**J**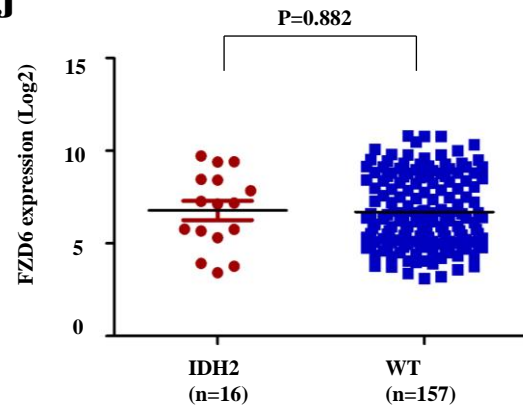

Supplement: Supplementary 3 — Supplementary Figure 3: (a) FZD6 expression differences between patients ≥60 years old and < 60 years old; (b) FZD6 expression differences between males and females; (c) FZD6 expression differences between WBC < 10 × 109/L and WBC ≥ 10 × 109/L; (d) FZD6 expression differences in patients who received different types of HSCT and no-HSCT; (e) FZD6 expression difference in FAB classifications; (f) FZD6 expression differences between patients had DMNT3A mutation (n = 42) or not (n = 131); (g) FZD6 expression differences between patients had RUNX1 mutation (n = 15) or not (n = 158). (h) FZD6 expression differences between patients had ASXL1 mutation (n = 3) or not (n = 170). (i) FZD6 expression differences between patients had IDH1 mutation (n = 17) or not (n = 156). (j) FZD6 expression differences between patients had DMNT3A mutation (n = 16) or not (n = 157). ∗P < 0.05; ∗∗P < 0.01; ∗∗∗P < 0.001. [file 9130958.f3.pdf]

**A**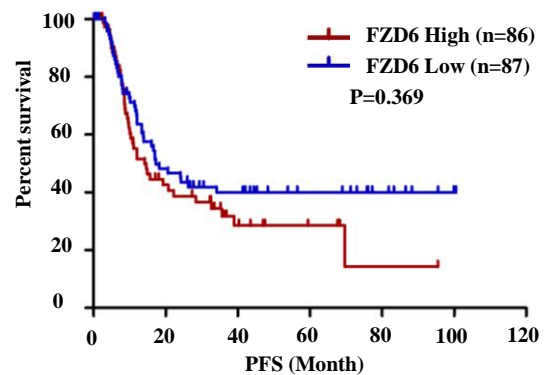**B**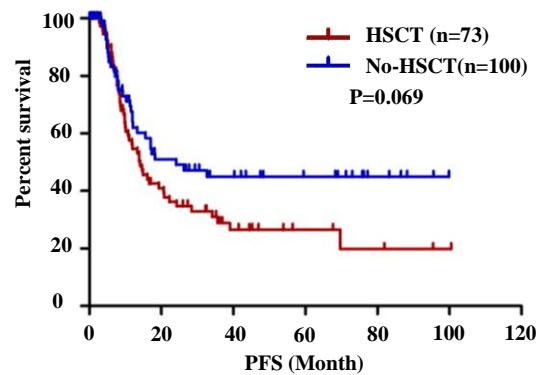**C**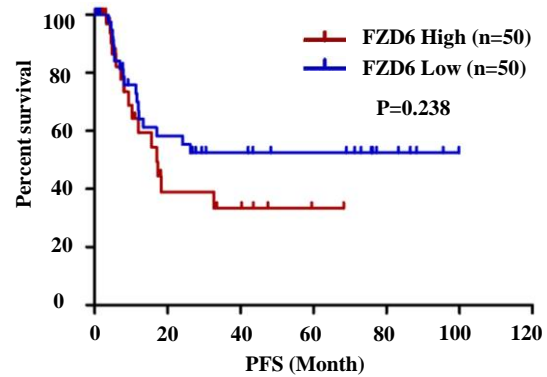**D**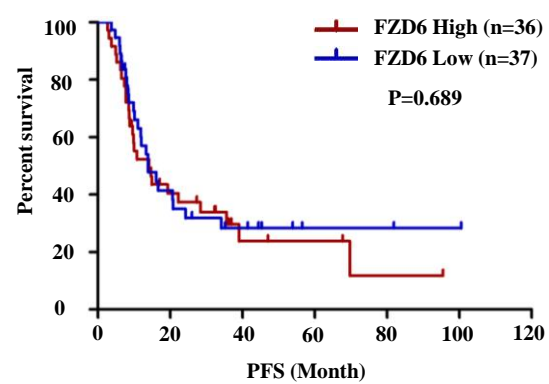

Supplement: Supplementary 4 — Supplementary Figure 4: (a) the association between FZD6 and EFS of AML patients; (b) the association between HSCT and EFS of AML patients; (c) the association between FZD6 and EFS of AML patients did not receive HSCT; (d) the association between FZD6 and EFS of AML patients who received HSCT. [file 9130958.f4.pdf]

A

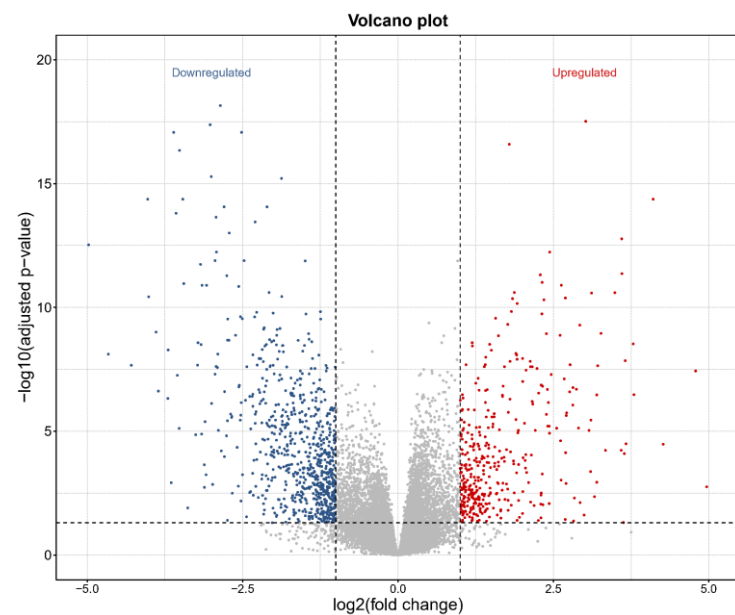

B

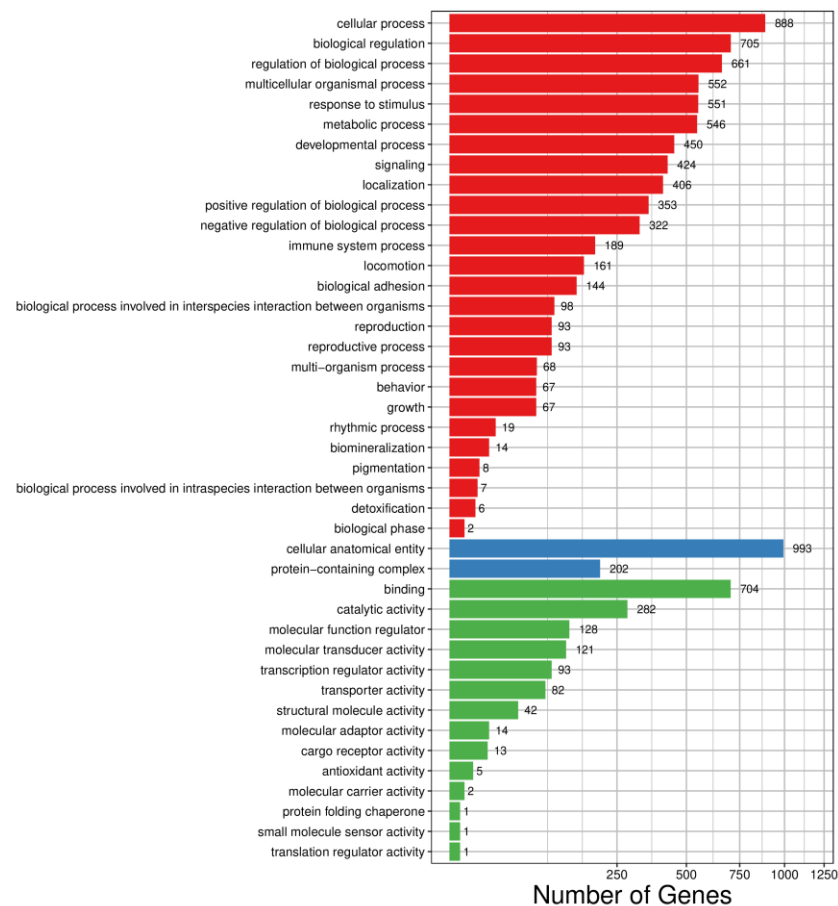

C

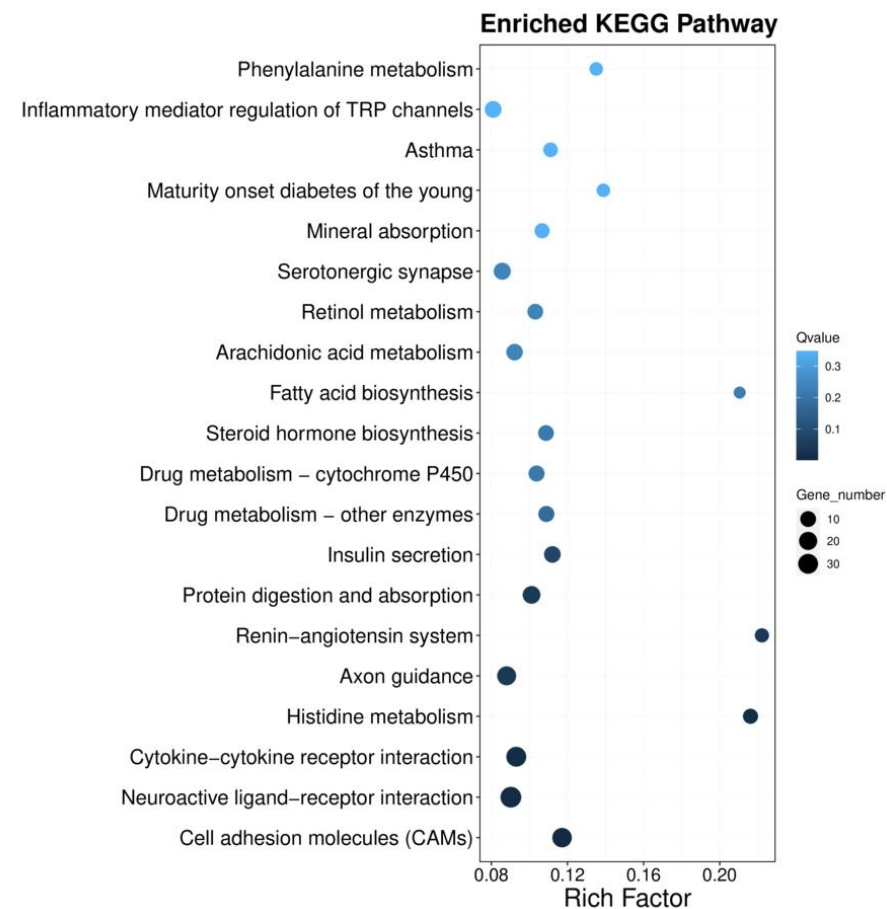

Supplement: Supplementary 5 — Supplementary Figure 5: (a) volcano plot of different gene-expression profiles between the high FZD6 and low FZD6 groups; (b) GO analysis associated with FZD6 expression; (c) top 20 KEGG pathway associated with FZD6 expression. [file 9130958.f5.pdf]

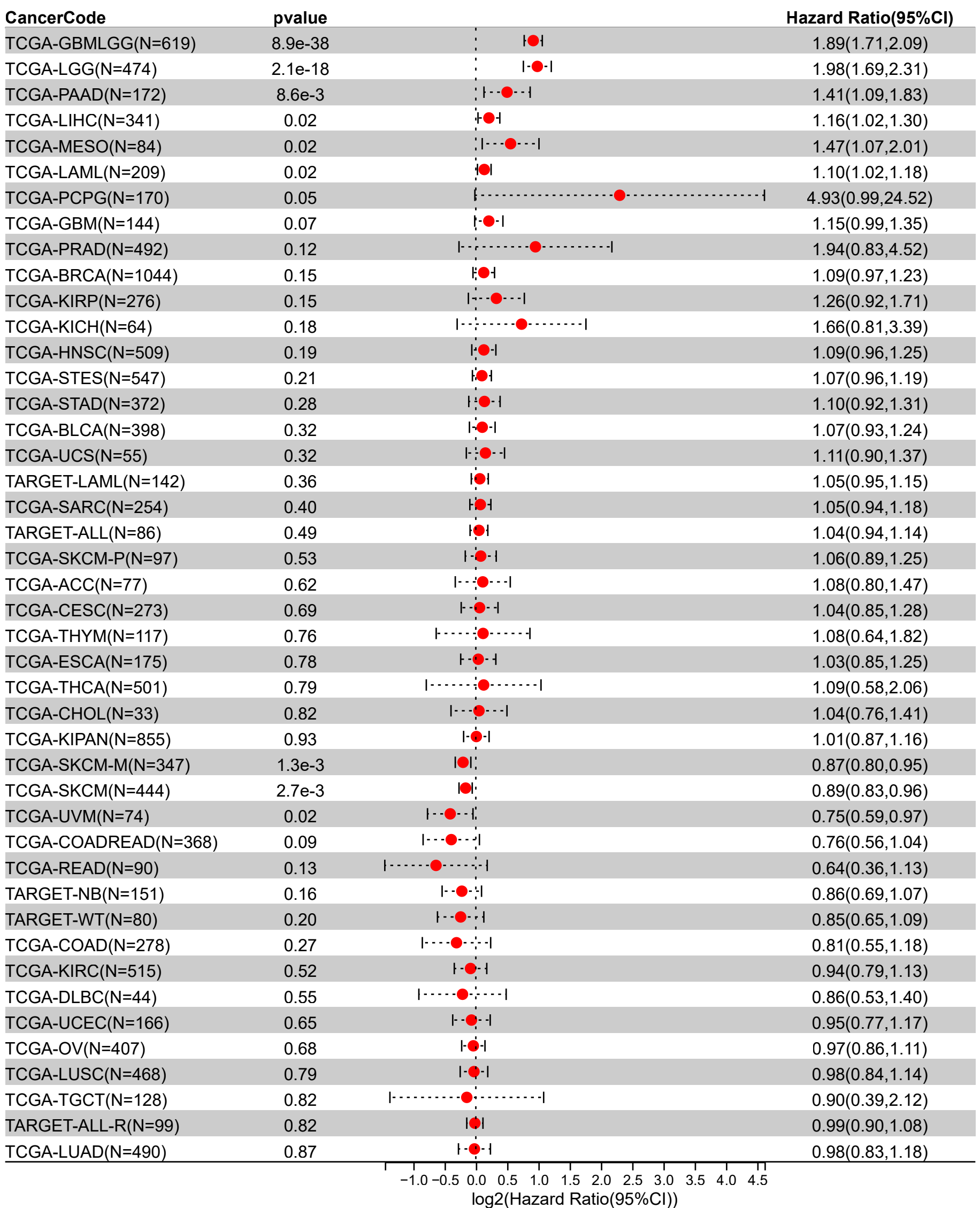

Supplement: Supplementary 6 — Supplementary Figure 6: the relationship between the expression of FZD6 and prognosis in pan-cancer. [file 9130958.f6.pdf]
