# Supplementary material for: Comprehensive Genomic Analysis for Identifying FZD6 as a Novel Diagnostic Biomarker for Acute Myeloid Leukemia
Source: Comput Math Methods Med. 2022 Nov 18;2022:9130958. doi: 10.1155/2022/9130958 (PMC9704059; doi:10.1155/2022/9130958)
Supplement: Supplementary 7 — Supplementary Table 1: coexpressed genes of FZD6 based on the cBioPortal dataset. [file 9130958.f7.docx]

**Supplemental table 1 Co-expressed genes of FZD6 based on the cBioPortal dataset.**

| **Correlated Gene** | **Cytoband** | **Spearman's Correlation** | **p-Value** | **q-Value** |
| --- | --- | --- | --- | --- |
| **MLLT3** | **9p21.3** | **0.575696** | **1.17E-16** | **1.42E-12** |
| **SPAG16** | **2q34** | **0.574325** | **1.44E-16** | **1.42E-12** |
| **AKAP6** | **14q12** | **0.558064** | **1.50E-15** | **9.89E-12** |
| **PRKCH** | **14q23.1** | **0.549488** | **4.92E-15** | **2.43E-11** |
| **MREG** | **2q35** | **0.534724** | **3.51E-14** | **1.39E-10** |
| **ATP8A1** | **4p13** | **0.528373** | **7.94E-14** | **2.61E-10** |
| **ARMCX5** | **Xq22.1** | **0.525071** | **1.21E-13** | **3.09E-10** |
| **KLHL6** | **3q27.1** | **0.524767** | **1.25E-13** | **3.09E-10** |
| **RAB39B** | **Xq28** | **0.522156** | **1.74E-13** | **3.81E-10** |
| **TRAF5** | **1q32.3** | **0.520637** | **2.10E-13** | **4.06E-10** |
| **MED12L** | **3q25.1** | **0.519367** | **2.45E-13** | **4.06E-10** |
| **CD34** | **1q32.2** | **0.519325** | **2.47E-13** | **4.06E-10** |
| **KLHL31** | **6p12.1** | **0.51708** | **3.25E-13** | **4.94E-10** |
| **EGF** | **4q25** | **0.514701** | **4.35E-13** | **6.13E-10** |
| **FAM124B** | **2q36.2** | **0.513808** | **4.85E-13** | **6.38E-10** |
| **CTHRC1** | **8q22.3** | **0.513177** | **5.23E-13** | **6.45E-10** |
| **LANCL1** | **2q34** | **0.510406** | **7.30E-13** | **8.48E-10** |
| **ABLIM1** | **10q25.3** | **0.506075** | **1.22E-12** | **1.34E-09** |
| **KDM4D** | **11q21** | **0.505277** | **1.34E-12** | **1.35E-09** |
| **MIR99AHG** | **21q21.1** | **0.505159** | **1.36E-12** | **1.35E-09** |
| **FAM169A** | **5q13.3** | **0.503129** | **1.73E-12** | **1.63E-09** |
| **PSD2** | **5q31.2** | **0.502606** | **1.84E-12** | **1.65E-09** |
| **PRKCQ** | **10p15.1** | **0.500658** | **2.31E-12** | **1.98E-09** |
| **ZIK1** | **19q13.43** | **0.500315** | **2.40E-12** | **1.98E-09** |
| **B4GALT6** | **18q12.1** | **0.499856** | **2.53E-12** | **2.00E-09** |
| **ABCB1** | **7q21.12** | **0.498925** | **2.82E-12** | **2.14E-09** |
| **GPRASP2** | **Xq22.1** | **0.497237** | **3.43E-12** | **2.50E-09** |
| **ARHGAP5** | **14q12** | **0.496447** | **3.75E-12** | **2.64E-09** |
| **GFI1B** | **9q34.13** | **0.495511** | **4.18E-12** | **2.84E-09** |
| **MN1** | **22q12.1** | **0.494704** | **4.58E-12** | **2.90E-09** |
| **C6ORF201** | **6p25.2** | **0.4946** | **4.63E-12** | **2.90E-09** |
| **IKZF2** | **2q34** | **0.494472** | **4.70E-12** | **2.90E-09** |
| **SIDT1** | **3q13.2** | **0.493397** | **5.31E-12** | **3.18E-09** |
| **CD274** | **9p24.1** | **0.491808** | **6.36E-12** | **3.65E-09** |
| **FAM30A** | **14q32.33** | **0.491329** | **6.71E-12** | **3.68E-09** |
| **ARHGAP5-AS1** | **14q12** | **0.490669** | **7.23E-12** | **3.86E-09** |
| **SYNJ2** | **6q25.3** | **0.490365** | **7.48E-12** | **3.89E-09** |
| **PRKD3** | **2p22.2** | **0.489536** | **8.21E-12** | **4.05E-09** |
| **GUCY1B1** | **4q32.1** | **0.487276** | **1.06E-11** | **5.01E-09** |
| **OPRK1** | **8q11.23** | **0.4872** | **1.07E-11** | **5.01E-09** |
| **SLC38A1** | **12q13.11** | **0.484965** | **1.36E-11** | **6.12E-09** |
| **CDCP1** | **3p21.31** | **0.484094** | **1.50E-11** | **6.47E-09** |
| **BEND4** | **4p13** | **0.484059** | **1.51E-11** | **6.47E-09** |
| **HPGD** | **4q34.1** | **0.480657** | **2.19E-11** | **9.00E-09** |
| **ZNF793** | **19q13.12** | **0.479245** | **2.55E-11** | **1.03E-08** |
| **MYT1** | **20q13.33** | **0.476058** | **3.60E-11** | **1.42E-08** |
| **CAPN14** | **2p23.1** | **0.474906** | **4.07E-11** | **1.54E-08** |
| **LRBA** | **4q31.3** | **0.474183** | **4.40E-11** | **1.64E-08** |
| **GSTA4** | **6p12.2** | **0.473464** | **4.75E-11** | **1.73E-08** |
| **TSPAN5** | **4q23** | **0.472815** | **5.09E-11** | **1.83E-08** |
| **PROM1** | **4p15.32** | **0.472434** | **5.30E-11** | **1.87E-08** |
| **PRKAB2** | **1q21.1** | **0.470929** | **6.21E-11** | **2.13E-08** |
| **SLC37A1** | **21q22.3** | **0.470697** | **6.36E-11** | **2.13E-08** |
| **SPRY1** | **4q28.1** | **0.470621** | **6.41E-11** | **2.13E-08** |
| **ZBED3-AS1** | **5q13.3** | **0.470534** | **6.47E-11** | **2.13E-08** |
| **TPD52** | **8q21.13** | **0.469309** | **7.36E-11** | **2.38E-08** |
| **POGLUT2** | **13q33.1** | **0.468797** | **7.77E-11** | **2.47E-08** |
| **PCP4L1** | **1q23.3** | **0.468068** | **8.38E-11** | **2.63E-08** |
| **F2RL1** | **5q13.3** | **0.467395** | **8.99E-11** | **2.77E-08** |
| **PLSCR4** | **3q24** | **0.466621** | **9.75E-11** | **2.96E-08** |
| **CD109** | **6q13** | **0.465807** | **1.06E-10** | **3.17E-08** |
| **APP** | **21q21.3** | **0.464567** | **1.21E-10** | **3.55E-08** |
| **ZMIZ1** | **10q22.3** | **0.463531** | **1.34E-10** | **3.86E-08** |
| **TTC23L** | **5p13.2** | **0.46347** | **1.35E-10** | **3.86E-08** |
| **SYNE1** | **6q25.2** | **0.462312** | **1.52E-10** | **4.21E-08** |
| **GBP4** | **1p22.2** | **0.462296** | **1.52E-10** | **4.21E-08** |
| **ECI2** | **6p25.2** | **0.462215** | **1.54E-10** | **4.21E-08** |
| **CDSN** | **6p21.33** | **0.461676** | **1.62E-10** | **4.33E-08** |
| **CYFIP2** | **5q33.3** | **0.461459** | **1.66E-10** | **4.37E-08** |
| **GUSBP5** | **4q31.21** | **0.460911** | **1.76E-10** | **4.54E-08** |
| **P2RY1** | **3q25.2** | **0.460833** | **1.77E-10** | **4.54E-08** |
| **ITGA9** | **3p22.2** | **0.460279** | **1.87E-10** | **4.74E-08** |
| **ZNF827** | **4q31.21-q31.22** | **0.459857** | **1.96E-10** | **4.89E-08** |
| **MTMR1** | **Xq28** | **0.459278** | **2.07E-10** | **5.05E-08** |
| **ADCY3** | **2p23.3** | **0.459062** | **2.12E-10** | **5.10E-08** |
| **BIVM** | **13q33.1** | **0.458499** | **2.24E-10** | **5.34E-08** |
| **GPR87** | **3q25.1** | **0.457882** | **2.39E-10** | **5.62E-08** |
| **PRR5L** | **11p13-p12** | **0.455827** | **2.94E-10** | **6.83E-08** |
| **VWA5A** | **11q24.2** | **0.454601** | **3.32E-10** | **7.63E-08** |
| **KRT73** | **12q13.13** | **0.453529** | **3.70E-10** | **8.39E-08** |
| **CNGA1** | **4p12** | **0.45316** | **3.84E-10** | **8.55E-08** |
| **NFATC2** | **20q13.2** | **0.453118** | **3.85E-10** | **8.55E-08** |
| **EVL** | **14q32.2** | **0.452269** | **4.19E-10** | **9.10E-08** |
| **ITGA6** | **2q31.1** | **0.451456** | **4.55E-10** | **9.76E-08** |
| **ENDOD1** | **11q21** | **0.450037** | **5.23E-10** | **1.10E-07** |
| **CA13** | **8q21.2** | **0.449778** | **5.37E-10** | **1.12E-07** |
| **DAAM1** | **14q23.1** | **0.448225** | **6.25E-10** | **1.27E-07** |
| **GLYATL1** | **11q12.1** | **0.447763** | **6.54E-10** | **1.30E-07** |
| **SLC9A7** | **Xp11.3** | **0.447708** | **6.58E-10** | **1.30E-07** |
| **APOBEC3F** | **22q13.1** | **0.44752** | **6.70E-10** | **1.31E-07** |
| **KMT2A** | **11q23.3** | **0.447254** | **6.87E-10** | **1.33E-07** |
| **ZNF462** | **9q31.2** | **0.447088** | **6.99E-10** | **1.33E-07** |
| **TAF4B** | **18q11.2** | **0.44705** | **7.01E-10** | **1.33E-07** |
| **DTNB** | **2p23.3** | **0.446069** | **7.72E-10** | **1.44E-07** |
| **DYRK3** | **1q32.1** | **0.444667** | **8.84E-10** | **1.62E-07** |
| **CRISPLD1** | **8q21.13** | **0.444211** | **9.24E-10** | **1.67E-07** |
| **MMRN1** | **4q22.1** | **0.442477** | **1.09E-09** | **1.93E-07** |
| **MYEF2** | **15q21.1** | **0.442458** | **1.09E-09** | **1.93E-07** |
| **WRNIP1** | **6p25.2** | **0.442352** | **1.11E-09** | **1.93E-07** |
| **OR51B4** | **11p15.4** | **0.441088** | **1.25E-09** | **2.13E-07** |
| **SV2A** | **1q21.2** | **0.440994** | **1.26E-09** | **2.13E-07** |
| **CCDC102B** | **18q22.1** | **0.440987** | **1.26E-09** | **2.13E-07** |
| **SCYL3** | **1q24.2** | **0.440692** | **1.30E-09** | **2.16E-07** |
| **PLCXD1** | **Xp22.33 and Yp11.32** | **0.440648** | **1.30E-09** | **2.16E-07** |
| **MMP16** | **8q21.3** | **0.440557** | **1.31E-09** | **2.16E-07** |
| **C2CD2** | **21q22.3** | **0.440053** | **1.38E-09** | **2.20E-07** |
| **NEDD4** | **15q21.3** | **0.439978** | **1.39E-09** | **2.20E-07** |
| **RAVER2** | **1p31.3** | **0.43996** | **1.39E-09** | **2.20E-07** |
| **RAG1** | **11p12** | **0.439605** | **1.44E-09** | **2.24E-07** |
| **ZNF256** | **19q13.43** | **0.439527** | **1.45E-09** | **2.24E-07** |
| **BAALC** | **8q22.3** | **0.439522** | **1.45E-09** | **2.24E-07** |
| **NPR3** | **5p13.3** | **0.439061** | **1.51E-09** | **2.32E-07** |
| **KRT72** | **12q13.13** | **0.438794** | **1.55E-09** | **2.36E-07** |
| **SHANK3** | **22q13.33** | **0.437957** | **1.68E-09** | **2.52E-07** |
| **ZNF677** | **19q13.42** | **0.436953** | **1.85E-09** | **2.75E-07** |
| **PRDM15** | **21q22.3** | **0.436775** | **1.88E-09** | **2.77E-07** |
| **BHLHB9** | **Xq22.1** | **0.436342** | **1.96E-09** | **2.87E-07** |
| **STAP2** | **19p13.3** | **0.435023** | **2.22E-09** | **3.22E-07** |
| **ZNF772** | **19q13.43** | **0.434194** | **2.40E-09** | **3.46E-07** |
| **ABCG1** | **21q22.3** | **0.433593** | **2.54E-09** | **3.61E-07** |
| **ZNF606** | **19q13.43** | **0.433447** | **2.57E-09** | **3.62E-07** |
| **ARHGEF9** | **Xq11.1** | **0.433306** | **2.61E-09** | **3.62E-07** |
| **CNKSR3** | **6q25.2** | **0.432356** | **2.85E-09** | **3.92E-07** |
| **ST6GAL1** | **3q27.3** | **0.432316** | **2.86E-09** | **3.92E-07** |
| **RAB9B** | **Xq22.2** | **0.431894** | **2.97E-09** | **4.05E-07** |
| **CEACAM1** | **19q13.2** | **0.43144** | **3.10E-09** | **4.19E-07** |
| **GRAMD1C** | **3q13.31** | **0.430957** | **3.24E-09** | **4.35E-07** |
| **ZNF418** | **19q13.43** | **0.430893** | **3.26E-09** | **4.35E-07** |
| **ZBTB8A** | **1p35.1** | **0.430752** | **3.31E-09** | **4.38E-07** |
| **GAS2** | **11p14.3** | **0.430308** | **3.44E-09** | **4.53E-07** |
| **BIN1** | **2q14.3** | **0.430165** | **3.49E-09** | **4.56E-07** |
| **KICS2** | **12q14.2** | **0.429373** | **3.75E-09** | **4.86E-07** |
| **CAPS2** | **12q21.1-q21.2** | **0.429331** | **3.77E-09** | **4.86E-07** |
| **DAPK1** | **9q21.33** | **0.429132** | **3.84E-09** | **4.92E-07** |
| **AMOT** | **Xq23** | **0.428564** | **4.05E-09** | **5.14E-07** |
| **VEZT** | **12q22** | **0.428418** | **4.10E-09** | **5.14E-07** |
| **MBD5** | **2q23.2** | **0.428397** | **4.11E-09** | **5.14E-07** |
| **TSPAN13** | **7p21.1** | **0.428371** | **4.12E-09** | **5.14E-07** |
| **CPT1A** | **11q13.3** | **0.428163** | **4.20E-09** | **5.21E-07** |
| **ADCY6** | **12q13.12** | **0.427213** | **4.58E-09** | **5.62E-07** |
| **SLC22A23** | **6p25.2** | **0.427199** | **4.58E-09** | **5.62E-07** |
| **DENND4C** | **9p22.1** | **0.427041** | **4.65E-09** | **5.67E-07** |
| **TSC22D1** | **13q14.11** | **0.42601** | **5.11E-09** | **6.19E-07** |
| **GSTO2** | **10q25.1** | **0.424842** | **5.68E-09** | **6.80E-07** |
| **SRSF8** | **11q21** | **0.424487** | **5.87E-09** | **6.98E-07** |
| **CARMIL1** | **6p22.2** | **0.424114** | **6.07E-09** | **7.18E-07** |
| **EHD3** | **2p23.1** | **0.421903** | **7.42E-09** | **8.61E-07** |
| **CD2AP** | **6p12.3** | **0.421743** | **7.52E-09** | **8.68E-07** |
| **HERC2** | **15q13.1** | **0.421136** | **7.94E-09** | **9.01E-07** |
| **CXXC5** | **5q31.2** | **0.421136** | **7.94E-09** | **9.01E-07** |
| **MAG** | **19q13.1** | **0.420841** | **8.16E-09** | **9.15E-07** |
| **MTURN** | **7p14.3** | **0.420714** | **8.25E-09** | **9.20E-07** |
| **MAST4** | **5q12.3** | **0.420647** | **8.30E-09** | **9.21E-07** |
| **ZNF154** | **19q13.43** | **0.420125** | **8.70E-09** | **9.59E-07** |
| **TIGD7** | **16p13.3** | **0.419847** | **8.92E-09** | **9.73E-07** |
| **TP53INP1** | **8q22.1** | **0.41984** | **8.92E-09** | **9.73E-07** |
| **ARHGAP8** | **22q13.31** | **0.419684** | **9.05E-09** | **9.77E-07** |
| **ZNF385D** | **3p24.3** | **0.419677** | **9.05E-09** | **9.77E-07** |
| **UBE4B** | **1p36.22** | **0.419546** | **9.16E-09** | **9.83E-07** |
| **MAP9** | **4q32.1** | **0.41942** | **9.26E-09** | **9.88E-07** |
| **MAPRE2** | **18q12.1-q12.2** | **0.418834** | **9.76E-09** | **1.04E-06** |
| **CD84** | **1q23.3** | **0.418663** | **9.91E-09** | **1.04E-06** |
| **CEACAM21** | **19q13.2** | **0.418403** | **1.01E-08** | **1.06E-06** |
| **GPLD1** | **6p22.3** | **0.418345** | **1.02E-08** | **1.06E-06** |
| **ABCA11P** | **4p16.3** | **0.418246** | **1.03E-08** | **1.06E-06** |
| **VIP** | **6q25.2** | **0.416867** | **1.16E-08** | **1.20E-06** |
| **RHOH** | **4p14** | **0.416655** | **1.18E-08** | **1.21E-06** |
| **MAP3K4** | **6q26** | **0.416496** | **1.20E-08** | **1.21E-06** |
| **CYYR1** | **21q21.3** | **0.416471** | **1.20E-08** | **1.21E-06** |
| **ENG** | **9q34.11** | **0.41647** | **1.20E-08** | **1.21E-06** |
| **CARD11** | **7p22.2** | **0.416352** | **1.22E-08** | **1.22E-06** |
| **DIPK1B** | **9q34.3** | **0.416185** | **1.23E-08** | **1.23E-06** |
| **HYKK** | **15q25.1** | **0.416023** | **1.25E-08** | **1.24E-06** |
| **C20ORF203** | **20q11.21** | **0.415956** | **1.26E-08** | **1.24E-06** |
| **TESPA1** | **12q13.2** | **0.415823** | **1.27E-08** | **1.25E-06** |
| **LRRC1** | **6p12.1** | **0.4158** | **1.28E-08** | **1.25E-06** |
| **BTRC** | **10q24.32** | **0.414407** | **1.44E-08** | **1.40E-06** |
| **CPS1** | **2q34** | **0.414313** | **1.46E-08** | **1.40E-06** |
| **ZNF704** | **8q21.13** | **0.414197** | **1.47E-08** | **1.41E-06** |
| **SYNM** | **15q26.3** | **0.414071** | **1.49E-08** | **1.42E-06** |
| **AHNAK2** | **14q32.33** | **0.413835** | **1.52E-08** | **1.43E-06** |
| **NLRC3** | **16p13.3** | **0.41345** | **1.57E-08** | **1.47E-06** |
| **TNXB** | **6p21.33-p21.32** | **0.413077** | **1.62E-08** | **1.51E-06** |
| **ASPRV1** | **2p13.3** | **0.412859** | **1.65E-08** | **1.52E-06** |
| **NHLRC2** | **10q25.3** | **0.412766** | **1.67E-08** | **1.52E-06** |
| **HMG20A** | **15q24.3** | **0.412688** | **1.68E-08** | **1.52E-06** |
| **SF3B3** | **16q22.1** | **0.412671** | **1.68E-08** | **1.52E-06** |
| **RRP1B** | **21q22.3** | **0.412502** | **1.71E-08** | **1.54E-06** |
| **SLC24A5** | **15q21.1** | **0.412206** | **1.75E-08** | **1.56E-06** |
| **ROBO4** | **11q24.2** | **0.412076** | **1.77E-08** | **1.57E-06** |
| **MICU3** | **8p22** | **0.41121** | **1.91E-08** | **1.67E-06** |
| **CRHBP** | **5q13.3** | **0.41119** | **1.91E-08** | **1.67E-06** |
| **PRKG2** | **4q21.21** | **0.410302** | **2.07E-08** | **1.80E-06** |
| **HSF5** | **17q22** | **0.410175** | **2.09E-08** | **1.81E-06** |
| **SERPINI1** | **3q26.1** | **0.410013** | **2.12E-08** | **1.82E-06** |
| **CNST** | **1q44** | **0.409973** | **2.13E-08** | **1.82E-06** |
| **BCAT1** | **12p12.1** | **0.409879** | **2.14E-08** | **1.82E-06** |
| **ELOVL6** | **4q25** | **0.409853** | **2.15E-08** | **1.82E-06** |
| **FAM95B1** | **9p11.2** | **0.409567** | **2.20E-08** | **1.85E-06** |
| **UTP25** | **1q32.2** | **0.408444** | **2.43E-08** | **2.02E-06** |
| **NEU3** | **11q13.4** | **0.408043** | **2.51E-08** | **2.08E-06** |
| **NYNRIN** | **14q12** | **0.407932** | **2.53E-08** | **2.09E-06** |
| **HCG4** | **6p22.1** | **0.407869** | **2.55E-08** | **2.10E-06** |
| **TDRKH** | **1q21.3** | **0.406859** | **2.78E-08** | **2.27E-06** |
| **KIF2A** | **5q12.1** | **0.406819** | **2.79E-08** | **2.27E-06** |
| **PIR** | **Xp22.2** | **0.40675** | **2.81E-08** | **2.27E-06** |
| **TPD52L1** | **6q22-q23** | **0.406177** | **2.95E-08** | **2.36E-06** |
| **APOBEC3G** | **22q13.1** | **0.406057** | **2.98E-08** | **2.38E-06** |
| **SCML4** | **6q21** | **0.405663** | **3.08E-08** | **2.44E-06** |
| **DNM3** | **1q24.3** | **0.405431** | **3.14E-08** | **2.47E-06** |
| **AMOTL1** | **11q21** | **0.405264** | **3.19E-08** | **2.49E-06** |
| **VN1R5** | **1q44** | **0.405081** | **3.24E-08** | **2.52E-06** |
| **KLF12** | **13q22.1** | **0.404914** | **3.28E-08** | **2.55E-06** |
| **HTT** | **4p16.3** | **0.404745** | **3.33E-08** | **2.58E-06** |
| **ACSL5** | **10q25.2** | **0.404601** | **3.37E-08** | **2.60E-06** |
| **SYNJ2BP** | **14q24.2** | **0.404555** | **3.38E-08** | **2.60E-06** |
| **SEPTIN11** | **4q21.1** | **0.404098** | **3.52E-08** | **2.68E-06** |
| **JAM2** | **21q21.3** | **0.403915** | **3.57E-08** | **2.71E-06** |
| **SLC16A10** | **6q21** | **0.403796** | **3.61E-08** | **2.73E-06** |
| **CNRIP1** | **2p14** | **0.402807** | **3.92E-08** | **2.96E-06** |
| **SYNGAP1** | **6p21.32** | **0.402568** | **4.00E-08** | **3.00E-06** |
| **PFKM** | **12q13.11** | **0.402293** | **4.10E-08** | **3.06E-06** |
| **GNG7** | **19p13.3** | **0.402001** | **4.20E-08** | **3.12E-06** |
| **ICAM5** | **19p13.2** | **0.40161** | **4.34E-08** | **3.19E-06** |
| **ZNF776** | **19q13.43** | **0.401602** | **4.34E-08** | **3.19E-06** |
| **ZNF204P** | **6p22.1** | **0.401264** | **4.47E-08** | **3.27E-06** |
| **DCLRE1A** | **10q25.3** | **0.400853** | **4.63E-08** | **3.36E-06** |
| **AGPAT5** | **8p23.1** | **0.400582** | **4.73E-08** | **3.41E-06** |
| **GYPC** | **2q14.3** | **0.400216** | **4.88E-08** | **3.50E-06** |
| **TAB2** | **6q25.1** | **0.400072** | **4.94E-08** | **3.53E-06** |
| **ATXN7L1** | **7q22.3** | **-0.40069** | **4.69E-08** | **3.39E-06** |
| **OVCA2** | **17p13.3** | **-0.40088** | **4.61E-08** | **3.36E-06** |
| **STAR** | **8p11.23** | **-0.40158** | **4.35E-08** | **3.19E-06** |
| **FUCA2** | **6q24.2** | **-0.40197** | **4.21E-08** | **3.12E-06** |
| **SPCS1** | **3p21.1** | **-0.40423** | **3.48E-08** | **2.66E-06** |
| **YKT6** | **7p13** | **-0.40548** | **3.13E-08** | **2.47E-06** |
| **TMX4** | **20p12.3** | **-0.40587** | **3.02E-08** | **2.41E-06** |
| **RNASEK** | **17p13.1** | **-0.40638** | **2.89E-08** | **2.33E-06** |
| **GDE1** | **16p12.3** | **-0.40711** | **2.72E-08** | **2.23E-06** |
| **STAC3** | **12q13.3** | **-0.4089** | **2.33E-08** | **1.95E-06** |
| **ATP6V0B** | **1p34.1** | **-0.40976** | **2.16E-08** | **1.83E-06** |
| **IRX5** | **16q12.2** | **-0.41013** | **2.10E-08** | **1.81E-06** |
| **C16ORF74** | **16q24.1** | **-0.41177** | **1.82E-08** | **1.60E-06** |
| **BUD31** | **7q22.1** | **-0.41221** | **1.75E-08** | **1.56E-06** |
| **ATG7** | **3p25.3** | **-0.41247** | **1.71E-08** | **1.54E-06** |
| **CATSPER1** | **11q13.1** | **-0.41273** | **1.67E-08** | **1.52E-06** |
| **DENND10** | **10q26.11** | **-0.41296** | **1.64E-08** | **1.52E-06** |
| **RNF135** | **17q11.2** | **-0.4137** | **1.54E-08** | **1.44E-06** |
| **OS9** | **12q13.3-q14.1** | **-0.41394** | **1.50E-08** | **1.43E-06** |
| **RAB5IF** | **20q11.23** | **-0.4153** | **1.33E-08** | **1.30E-06** |
| **ASL** | **7q11.21** | **-0.41864** | **9.93E-09** | **1.04E-06** |
| **APOC2** | **19q13.32** | **-0.42088** | **8.13E-09** | **9.15E-07** |
| **CT45A1** | **Xq26.3** | **-0.42136** | **7.78E-09** | **8.93E-07** |
| **PLAUR** | **19q13** | **-0.42292** | **6.76E-09** | **7.90E-07** |
| **MAP6D1** | **3q27.1** | **-0.42391** | **6.18E-09** | **7.27E-07** |
| **AZU1** | **19p13.3** | **-0.42518** | **5.51E-09** | **6.63E-07** |
| **DENND1A** | **9q33.3** | **-0.43338** | **2.59E-09** | **3.62E-07** |
| **TP53INP2** | **20q11.22** | **-0.43357** | **2.54E-09** | **3.61E-07** |
| **RNASE3** | **14q11.2** | **-0.43821** | **1.64E-09** | **2.48E-07** |
| **TBX1** | **22q11.21** | **-0.44021** | **1.36E-09** | **2.20E-07** |
| **RBKS** | **2p23.2** | **-0.44025** | **1.35E-09** | **2.20E-07** |
| **LRMDA** | **10q22.2-q22.3** | **-0.44144** | **1.21E-09** | **2.09E-07** |
| **HAL** | **12q23.1** | **-0.4425** | **1.09E-09** | **1.93E-07** |
| **DPRXP4** | **17q11.2** | **-0.4455** | **8.15E-10** | **1.50E-07** |
| **PLPPR3** | **19p13.3** | **-0.44632** | **7.53E-10** | **1.42E-07** |
| **MRPL33** | **2p23.2** | **-0.44804** | **6.36E-10** | **1.28E-07** |
| **CST3** | **20p11.21** | **-0.44887** | **5.87E-10** | **1.21E-07** |
| **ZNHIT1** | **7q22.1** | **-0.45057** | **4.96E-10** | **1.05E-07** |
| **RNASE2** | **14q11.2** | **-0.4526** | **4.06E-10** | **8.91E-08** |
| **PPP1R27** | **17q25.3** | **-0.45965** | **2.00E-10** | **4.93E-08** |
| **RAC1** | **7p22.1** | **-0.462** | **1.57E-10** | **4.25E-08** |
| **HOMER3** | **19p13.11** | **-0.47491** | **4.07E-11** | **1.54E-08** |
| **RPA4** | **Xq21.33** | **-0.48112** | **2.08E-11** | **8.74E-09** |
| **CFD** | **19p13.3** | **-0.48508** | **1.35E-11** | **6.12E-09** |
| **BTG1** | **12q21.33** | **-0.48974** | **8.02E-12** | **4.05E-09** |
| **PARL** | **3q27.1** | **-0.49165** | **6.47E-12** | **3.65E-09** |
